# (−)-Complanine, an inflammatory substance of marine fireworm: a synthetic study

**DOI:** 10.3762/bjoc.5.12

**Published:** 2009-04-16

**Authors:** Kazuhiko Nakamura, Yu Tachikawa, Daisuke Uemura

**Affiliations:** 1 Department of Biosciences and Informatics, Keio University 3-14-1 Hiyoshi Yokohama 223-8622, Japan; 2 Graduate School of Science, Nagoya University Furo-cho Chikusa Nagoya 464-8566, Japan

**Keywords:** chiral synthon, complanine, inflammatory substance, marine fireworm, total synthesis

## Abstract

The synthesis of (−)-complanine, an inflammatory substance of *Eurythoe complanata*, was accomplished by a “chiral synthon” approach. The absolute configuration of this molecule was determined to be *R*.

## Introduction

**Figure 1:**
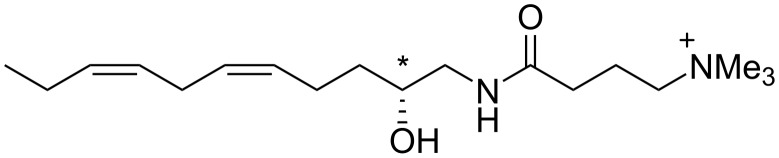
Structure of (*R*)-(−)-complanine.

**Figure 2:**
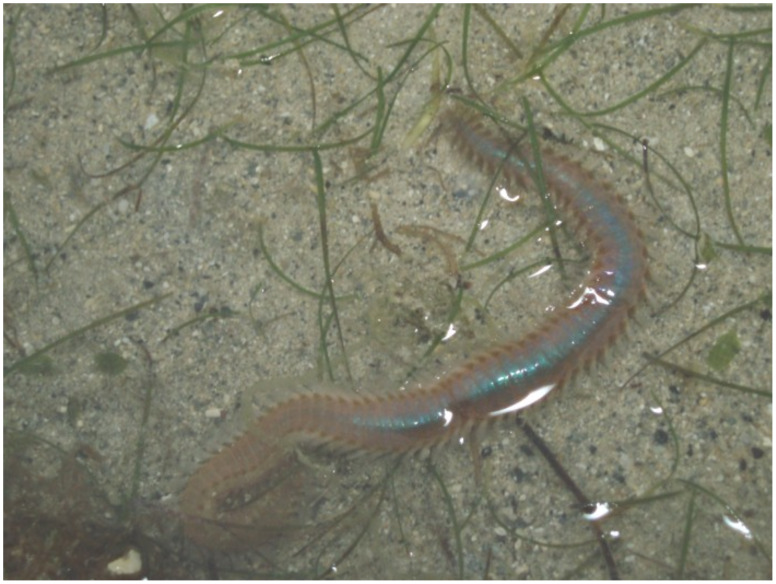
Marine fireworm *Eurythoe complanata* (body length 10 cm).

Toxic marine annelids were first referred to in the literature as “sea scolopendra” in *de Materia Medica* (A.D. 50) by Dioscorides, a physician of the Roman Empire [1]. The marine animals, which are commonly known as “fireworms”, are dangerous to humans, as careless handling with bare hands can result in serious dermatitis. However the actual toxic substance of these animals has remained unknown. We recently isolated a novel amphipathic substance, named complanine (Figure 1), from an amphinomid polychaete, *Eurythoe complanata* (Figure 2). Complanine has been identified as an inflammatory substance by bioassay-guided separations; and the substance is thought to be used as part of the animal’s defense system. In a previous study the molecular mechanism of inflammation by the action of complanine was examined, and its activation of protein kinase C (PKC) in the presence of Ca^2+^ and 12-*O*-tetradecanoylphorbol 13-acetate (TPA) has been proved. These results suggest that complanine can bind PKC at the same site as phosphatidylserine, a co-activation factor with Ca^2+^ and TPA. It is known that signal transduction leads to an inflammation mediator TNF-α and its downstream signal molecules, which occurs by phosphorylation through the action of PKC; thus, the biological properties of complanine can be understood as controlling this cascade [2].

From a structural perspective, complanine possesses amphipathic properties due to its characteristic unsaturated carbon chain and a γ-aminobutyric acid (GABA)-derived trimethylammonium substructure. Natural complanine shows negative optical rotation ([*α*]_D_^25^ = −10.0 (*c* 1.0, H_2_O)), but the configuration of the hydroxy-substituted carbon atom has not been revealed because derivatization to determine the absolute configuration failed due to the lack of availability of the natural product. In this study, the absolute structure of complanine was unambiguously determined by means of synthetic methodology by a “chiral synthon” approach. Related amino alcohols possessing olefins from marine natural resources have been identified [3–4], but synthetic studies of these compounds have not been reported.

## Results and Discussion

**Scheme 1:**
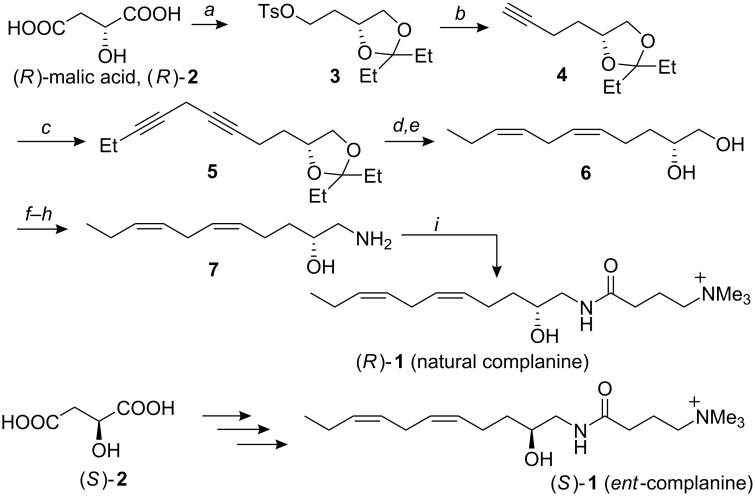
Total synthesis of complanine. Keys: *a*) 1. BH_3_·SMe_2_ (71%); 2. *cat.* TsOH, Et_2_CO (59%); 3. TsCl, pyridine (80%) [5–6]; *b*) lithium acetylide ethylenediamine complex (1.2 equiv), DMSO, rt, 3 h (51%); *c*) 1-iodopent-2-yne (2.0 equiv), EtMgBr (1.6 equiv), CuI (cat.), THF, 0 °C to rt, 12 h (43%); *d*) H_2_, Lindlar catalyst, EtOH, rt, 30 min; *e*) AcOH, H_2_O, rt, 12 h (43% in 2 steps); *f*) MsCl (1.1 equiv), pyridine, CH_2_Cl_2_, 0 °C, 2 h; *g*) NaN_3_ (4.0 equiv), DMF, 80 °C, 11 h (79% in 2 steps); *h*) PPh_3_ (1.0 equiv), THF, H_2_O, rt, 12 h (78%); *i*) *N*-[4-(trimethylammonio)butyryloxy]succinimide iodide (see text and [9]) (2.0 equiv), MeOH, rt, 18 h (44%).

Our synthesis started from the known compound **3** [5–6] that could be derived from (*R*)-malic acid, (*R*)-**2**, in three steps (1. BH_3_·SMe_2_; 2. *cat.* TsOH, Et_2_CO; 3. TsCl, pyridine) (Scheme 1). The resultant tosylate **3** was treated with lithium acetylide ethylenediamine complex to give the terminal acetylene **4** in 51% yield [7]. The bromomagnesium salt of **4** generated with EtMgBr was successively treated with 1-iodopent-2-yne in the presence of CuI to give the corresponding diyne compound **5** in 43% yield [8]. The partial reduction was achieved by using Lindlar catalyst to give the desired *Z* olefin, which was then subjected to acidic deprotection to afford the diol **6** in 43% yield (2 steps). The primary alcohol was converted into the azide *via* the mesylate (79%), which was then successfully converted into the corresponding amino alcohol **7** (78%). From a spectral perspective, the amino alcohol **7** was identical to the degradation product of natural complanine (NMR, MS and *R_f_* value of TLC).

The activated ester (hydroxysuccinimide ester) of 4-(trimethylammonio)butanoate was synthesized from the commercially available γ-aminobutyric acid (GABA) in two steps (1. MeI, NaHCO_3_, MeOH, rt, 24 h; 2. HOSu; DCC, CH_3_CN, rt, 24 h) [9]. A reaction occurred between the amino alcohol and the activated ester (2.0 equiv) in MeOH to give the desired (−)-complanine in 44% yield. The synthesized product was identical to the natural material in all its spectral data, including optical rotation ([*α*]_D_^20^ = −9.9 (*c* 0.12, H_2_O)). The configuration of the hydroxy-substituted carbon atom was determined to be *R*. The configuration is comparable to that of the related compound, obscuraminol, isolated from an ascidian from Tarifa Island, Spain. Obscuraminol possesses a *vic*-amino alcohol and an unsaturated carbon chain; its absolute configuration (of the OH adjacent carbon atom) is *R* [4]. The similarity of the structures suggests a close relationship in their biosynthetic pathways. It can be hypothesized that complanine is biosynthesized from glycine, based on comparison with serine- or alanine-derived natural products [3–4,10–11].

The enantiomer of the natural product, (+)-complanine, was also successfully synthesized from the corresponding (*S*)-malic acid, (*S*)-**2**, in 10 steps, including coupling with 4-(trimethylammonio)butanoate. (*S*)-**1** (*ent*-complanine) showed positive optical rotation ([*α*]_D_^23^ = 11.1 (*c* 0.65, H_2_O)); which was in reasonably good agreement with the absolute configuration of the natural product. The biological activities of both enantiomers were examined, but no significant difference between them was observed based on the inflammatory activity on a mouse foot pad. Detailed biological properties (for example, PKC activation) of both enantiomers are under consideration at the present time.

In conclusion, (−)-complanine was successfully synthesized from (*R*)-malic acid by acetylene coupling and catalytic hydrogenation as key steps. The absolute configuration of the natural product was determined to be *R*.

## Experimental

**Synthesis of alkyne 4:** To a solution of tosylate (**3**, 3.00 g, 9.1 mmol) in DMSO (10 ml), a lithium acetylide ethylene diamine complex (1.00 g, 11.0 mmol) was added under nitrogen atmosphere. After stirring for 3 h, an extractive workup and column chromatography (SiO_2_, hexane/ethyl acetate 99 : 1) gave the desired alkyne **4** as a pale yellow oil (850 mg, 51%). **4:** [*α*]_D_^26^ +2.1 (*c* 1.0, CHCl_3_); HRMS (ESI) calcd for C_11_H_18_O_2_Na [(M + Na)^+^] 205.1204, found 205.1201; ^1^H NMR (400 MHz, CDCl_3_) *δ* 4.14 (1H, m), 4.04 (1H, dd, *J* = 6.2, 7.6 Hz), 3.48 (1H, t, *J* = 7.6 Hz), 2.27 (2H, m), 1.91 (1H, t, *J* = 2.8 Hz), 1.78 (1H, m), 1.69 (1H, m), 1.57 (4H, m), 0.84 (3H + 3H, t, *J* = 7.6 Hz, overlapped).

**Synthesis of diyne 5:** To a solution of the alkyne (**4**, 445 mg, 2.45 mmol) in THF (2.0 ml), 1.6 M solution of ethylmagnesium bromide (Aldrich, 2.45 ml, 3.92 mmol) was added under N_2_, and the resulting solution was stirred at ambient temperature. After 15 min, copper(I) iodide (2.0 mg, catalytic) was added, and the solution was stirred for additional 12 h. The solution was cooled to 0 °C, and 1-iodopent-2-yne (950 mg, 4.90 mmol) was added to the reaction mixture and gradually warmed to room temperature. After stirring for 12 h, an extractive workup and column chromatography (SiO_2_, hexane/ethyl acetate 98 : 2 to 95 : 5) gave the desired diyne compound **5** as a pale yellow oil (258 mg, 43%). **5:** [*α*]_D_^26^ +2.7 (*c* 0.26, CHCl_3_); HRMS (ESI) calcd for C_16_H_24_O_2_Na [(M + Na)^+^] 271.1674, found 271.1696; ^1^H NMR (600 MHz, CDCl_3_) *δ* 4.17 (1H, m), 4.09 (1H, dd, *J* = 6.0, 7.6 Hz), 3.52 (1H, dd, *J* = 7.6, 7.6 Hz), 3.11 (2H, t, *J* = 2.2 Hz), 2.29 (2H, m), 2.17 (2H, m), 1.80 (1H, m), 1.69 (1H, m), 1.62 (4H, m), 1.12 (3H, t, *J* = 7.6 Hz), 0.89 (3H + 3H, t, *J* = 7.4 Hz, overlapped).

**Synthesis of diol 6:** To the solution of the diyne **5** (103 mg, 0.410 mmol) in ethanol (4.0 ml), Lindlar catalyst (206 mg) was added. The mixture was stirred under hydrogen atmosphere for 30 min. After filtration of the catalyst, the concentrated residue was dissolved in an AcOH/H_2_O (4.0 : 3.5 ml) mixture and then stirred for 13 h. The concentrated residue was subjected to column chromatography (SiO_2_, hexane/ethyl acetate 1 : 1) to give the desired diol **6** as a colorless oil. **6:** [*α*]_D_^20^ −2.0 (*c* 0.40, CHCl_3_); HRMS (ESI) calcd for C_11_H_20_O_2_Na [(M + Na)^+^] 207.1361, found 207.1346; ^1^H NMR (600 MHz, CD_3_OD) *δ* 5.24–5.35 (4H, m), 3.53 (1H, br, s), 3.41 (2H, m), 2.77 (2H, t, *J* = 5.2 Hz), 2.17 (1H, m), 2.12 (1H, m), 2.03 (2H, m), 1.50 (1H, m), 1.39 (1H, m), 0.92 (3H, t, *J* = 7.7 Hz); ^13^C NMR (CD_3_OD) *δ* 132.6, 130.4, 129.6, 128.4, 72.7, 67.4, 34.4, 26.4, 24.3 21.4, 14.6.

**Synthesis of amino alcohol 7 via an azide:** To the solution of the diol (**6**, 16.2 mg, 88 μmol) in dichloromethane (2.0 ml), pyridine (1.2 ml) and mesyl chloride (11.1 mg, 97 μmol) were added. After stirring for 2 h, the extractive workup was carried out to give a colorless oil (24.2 mg). This crude material was successively dissolved in DMF (0.4 ml), and sodium azide (23 mg, 330 μmol) was then added. The reaction mixture was maintained at 80 °C for 11 h. An extractive workup and preparative TLC (SiO_2_, ethyl acetate) gave the desired azide as a colorless oil (14.6 mg, 79% in 2 steps). IR (CHCl_3_): 2105 cm^−1^.

The solution of the resultant azide (14.6 mg, 70 μmol) in THF (1.0 ml), H_2_O (25 μl) and triphenylphosphane (18.3 mg, 70 μmol) were added. After stirring for 12 h, the reaction mixture was concentrated, and the desired product (10.0 mg, 78%) was obtained by chromatography on SiO_2_ (eluent: CHCl_3_/MeOH/H_2_O 10 : 5 : 1) as a colorless oil. **7:** HRMS (ESI) calcd for C_11_H_22_NO [(M + H)^+^] 184.1701, found 184.1707; ^1^H NMR (600 MHz, CD_3_OD) *δ* 5.25–5.37 (4H, br m), 3.75 (1H, br s), 2.96 (1H, dd, *J* = 3.1, 12.4 Hz), 2.79 (2H, t, *J* = 6.6 Hz), 2.73 (1H, dd, *J* = 9.6, 12.4 Hz), 2.23 (1H, m), 2.16 (1H, m), 2.06 (2H, m), 1.49 (2H, m), 0.95 (3H, t, *J* = 7.6 Hz); ^13^C NMR (150 MHz, CD_3_OD) *δ* 131.4, 129.0, 128.3, 127.0, 71.4, 46.9, 34.5, 25.0, 23.0, 20.1, 13.3.

**Synthesis of (*R*)-complanine, (*R*)-1:** To the solution of the amino alcohol **7** (1.8 mg, 9.9 μmol) in MeOH (150 μl), *N*-[4-(trimethylammonio)butyryloxy]succinimide iodide [9] (5.5 mg, 20 mmol) in MeOH (150 μl) was added. The reaction mixture was stirred for 18 h. The resultant mixture was concentrated, and the residue was purified by column chromatography (SiO_2_, CHCl_3_/MeOH/H_2_O/AcOH 10 : 5 : 1 : 0.06) to give the synthetic complanine (1.4 mg, 44%) as a colorless oil. (*R*)-**1** (synthetic complanine): HRMS (ESI) calcd for C_18_H_35_N_2_O_2_^+^ [(M)^+^] 311.2693, found 311.2698; [*α*]_D_^20^ = −9.9 (*c* 0.12, H_2_O); ^1^H NMR (600 MHz, D_2_O) *δ* 5.31–5.37 (4H, br m), 3.64 (1H, br m), 3.22 (2H + 1H, m), 3.09 (1H, dd, *J* = 6.9, 13.8 Hz), 3.02 (9H, s), 2.43 (2H, t, *J* = 6.5 Hz), 2.28 (2H, t, *J* = 7.6 Hz), 2.08 (2H, m), 1.98 (2H, m), 1.41 (2H, m), 0.84 (3H, t, *J* = 7.6 Hz); ^13^C NMR (150 MHz, D_2_O) *δ* 180.9, 174.3, 129.6, 129.1, 127.6, 69.5, 65.5, 52.9 (3C), 45.9, 33.8, 31.7, 25.2, 23.3, 20.2, 18.8, 13.8.

**Synthesis of (*S*)-complanine, (*S*)-1:** The enantiomer of natural complanine was also synthesized from (*S*)-malic acid. (*S*)-**1** (*ent*-complanine): [*α*]_D_^23^ = 11.1 (*c* 0.65, H_2_O).
